# Healthcare workers’ delivery of adolescent responsive sexual and reproductive healthcare services: an assessment in Plateau state, Nigeria

**DOI:** 10.1186/s12905-023-02288-1

**Published:** 2023-03-25

**Authors:** Esther Awazzi Envuladu, Karlijn Massar, John de Wit

**Affiliations:** 1 Department of Community Medicine, College of Health Sciences, University of Jos, Jos, P.M.B 2084, Nigeria; 2 Department of Interdisciplinary Social Science, Utrecht University, P.O. Box 80140, Utrecht, 3508 The Netherlands; 3 Department of Work & Social Psychology, Maastricht University, P.O. Box 616, Maastricht, 6200 The Netherlands

**Keywords:** Sexual, Reproductive, Services, Policy, Adolescent-responsiveness, Nigeria

## Abstract

**Background:**

Adolescents should have access to high quality and responsive sexual and reproductive health, however, it is unclear to what extent the national policy on health and development of adolescent is implemented by health care workers in Plateau State. This study assessed the general availability of sexual and reproductive health services, the delivery of responsive adolescent sexual and reproductive health services and health care worker?s understanding of what constitutes adolescent responsive sexual and reproductive health services.

**Methods:**

Using a cross sectional design, we interviewed 409 health care workers selected through a multistage sampling technique, across six Local Government Areas of Plateau State, Nigeria using an interviewer-administered survey questionnaire.

**Results:**

The most available sexual and reproductive health services was antenatal and delivery care (69.2%), contraception 25.9% and 14.9% reported post abortion care. Only 1.2% indicated the availability of the four recommended essential sexual and reproductive health services (counselling/information provision, provision of contraceptives, testing/treatment for sexually transmitted infection (STI) /HIV and post abortion care) in their facilities. Little over half (58.4%) felt their facilities were adequate in meeting the sexual and reproductive health needs of adolescent and this was associated with delivery of post abortion care (AOR=3.612; CI=1.886-6.917; p = .001) and providing sexual and reproductive health services to adolescents without parental consent (AOR=3.612; CI=1.886-6.917; p = .001). Most health care workers had poor understanding of adolescent responsiveness of sexual and reproductive health services, understanding better among health workers who provided services without parental consent and in a separate room for privacy and confidentiality.

**Conclusion:**

We conclude that adolescent sexual and reproductive health services is not yet as stipulated in the national policy in Plateau State, Nigeria and in general, health workers have poor understanding of what it means to provide adolescent-responsive services.

**Supplementary Information:**

The online version contains supplementary material available at 10.1186/s12905-023-02288-1.

## Introduction

Access to comprehensive sexual and reproductive health (SRH) information and other services is a basic right of adolescents, as acknowledged at the 4th International Conference on Population and Development (ICPD) in Cairo in 1994, and enshrined in the Rights of the Child [1–3]. These international agreements are critical to ensure the attainment of a high standard of SRH for all, leaving no one behind [1, 4]. In recognition of the SRH needs of adolescents, Nigeria, like many other countries, is committed to ensuring the provision of adolescent sexual and reproductive health services (SRHS) to prevent unwanted SRH outcomes, such as unintended pregnancy, unsafe abortion, sexually transmitted infections (STI) and HIV [5–7]. As part of this commitment, and to adhere to international agreements, a national policy on the health and development of adolescent and young people was developed in 2007, and recently revised in 2019 [8–10]. This policy specifically aims to increase the availability, accessibility, delivery of responsive SRH information and services to meet the needs of adolescents and avoid preventable death [7].

According to the policy, adolescents should be provided scientifically accurate SRH information on sexuality, menstrual hygiene, prevention of unintended pregnancy and prevention of STI and HIV [8, 9]. Furthermore, healthcare workers (HCWs) are expected to provide counselling to adolescents on the full range of family planning methods and offer contraceptive services that are safe, affordable and acceptable to them to prevent unintended pregnancy [8, 9]. Adolescents should also be offered counselling on safe sex practices to prevent STI/HIV infection, as well as screening and treatment for STI and HIV where needed. For adolescents who are pregnant, the policy stipulates that antenatal, delivery and post-natal services should be provided, to ensure safe delivery and quality care for the mother and the baby. Abortion is not legal in Nigeria but high rates of unsafe illegal abortions are reported; therefore, the policy requires that post-abortion services should be provided for adolescents who experience abortion complications, either from self-induced or spontaneous abortion [9, 11].

All SRHS should be delivered in a non-judgmental manner and in an environment that respects the rights and privacy of adolescents (e.g, without parents) [9, 11–13]. Such delivery of unrestricted SRHS that meet the needs of adolescents in an environment that is supportive and respects the privacy and confidentiality of adolescents is considered *adolescent responsive* [14–16]. One of the initial steps taken by the Nigerian government to provide adolescent responsive SRHS was the creation of youth-friendly health centres, which were designed just for adolescents and young people and operated as stand-alone centres to address the existing challenge of poor access and utilization of SRHS [17–20].

To ensure access to SRHS and improve adolescent SRH health seeking, adolescent health services were integrated into the primary healthcare (PHC) centres, which are found in all communities and are often closer to where adolescents spend most of their time [9, 15]. We assessed the availability and accessibility of adolescent sexual and reproductive health services (ASRHS) in PHCs in Plateau State [21–23] and found that the range of services included in the policy were not available in most of the facilities surveyed. Those facilities that did provide SRHS were mainly adult focused (e.g., maternal healthcare) and not adolescent-centred [21, 22]. Moreover, we showed that adolescents did not seek SRH care in health facilities and were unwilling to utilize the health facilities for SRH care – even when they were in need [21, 23].

While geographical and financial constraints may impede adolescent’s utilization of SRHS, prior research has also shown that the negative attitudes of HCW and their non-responsiveness to the needs of adolescents, impedes adolescents’ SRH care seeking [24–27]. Also, while healthcare workers’ negative attitudes may be a reason for the hesitancy of some healthcare worker to provide ASRHS, others may be constrained by lack of SRH resources and inadequate understanding of adolescent responsive SRHS [28–30, and 31]. The aim of this study was to assess the general availability of SRHS and the delivery of ASRHS more specifically in primary care facilities from the perspective of HCW. We also assessed the views of HCW regarding their facilities meeting the SRH needs of adolescents and their understanding of what constitutes adolescent responsive SRHS. Furthermore, we examined the extent to which demographic and professional characteristics of HCW were associated with their self-reported delivery of ASRHS. Covariates of the extent to which HCW perceived their facilities to meet the SRH needs of adolescents and their understanding of adolescent responsive SRHS were also assessed.

## Methods

### Design and participants

A cross sectional survey was conducted among healthcare workers in PHC facilities in six selected Local Government Areas (LGAs) in Plateau State, north-central Nigeria. Every LGA has an average of 34 PHCs that provide basic health services, which include health promotion/education and treatment of common illnesses, in addition to providing SRHS. A diversity of HCWs provided consultation services at PHC, including nurses, community health extension workers, environmental health officers, laboratory technicians and volunteers. Although consulting health services at PHC should be delivered by doctors, nurses, midwives or medical assistants, due to a shortage of these types of staff members in PHC in Plateau State, workers from other cadres are also tasked with providing consulting health services.

The participants were recruited through a multistage sampling technique. We began by selecting six LGAs from the 17 LGAs in Plateau State. All the 230 PHC facilities across these six selected LGAs were included in the study. Of an estimated 690 HCWs in the selected PHCs, those who never provided any consulting services were excluded, resulting in a total of 446 HCWs who were eligible for participation in the study. Out of these, 409 (91.7%) HCWs completed the survey. All HCWs provided informed consent before commencing with the survey.

### Measures

Eight trained volunteer resident doctors from Jos University Teaching Hospital (JUTH) administered a self-report questionnaire through face-to-face interviews. Questions about SRHS available and provided to adolescents reflected the national policy on adolescent and young people [7]. Questions about adolescent responsiveness were derived from the literature [14]. The questionnaire was pretested and checked for any ambiguities and mistakes among 40 HCWs who did not participate in this study. Ambiguities and mistakes were corrected before administering the survey to the participants in this study.

*Demographic and professional characteristics*: The demographic and professional information collected included: age, sex, marital status and education, specialty of healthcare workers, years of working and if trained on adolescent SRH.

*Availability of SRHS*: The HCWs were asked about the SRHS generally available in their health facilities: contraceptives (yes/no), pregnancy testing (yes/no), STI/HIV testing (yes/no), STI/HIV treatment (yes/no), post abortion care (yes/no) and antenatal and delivery care (yes/no).

*Provision of ASRHS*: To assess if SRHS were provided to adolescents as stipulated in the national policy, HCW were asked if they provided the following SRHS to adolescents: SRH counselling (Yes/No), contraceptives (Yes/ No), STI treatment (Yes/No) and post abortion care (Yes/No). They were also asked if, as also stipulated in the national policy, ASRHS were provided in separate/private rooms to ensure privacy and confidentiality (Yes/No) and were provided without parental consent (Yes/No).

#### Perceived adequacy of facilities in meeting adolescents’ SRHS needs

HCWs were asked if they thought their facilities had adequate space, equipment and commodities to provide SRH meeting the needs of adolescents (Yes/No).

*Understanding of adolescent responsiveness of SRHS*: HCW were asked; ‘SRHS are adolescent-responsive when….’ and choose one or more of the following response options: 1) all SRHS are made available and accessible to adolescents, 2) the services are provided free or at affordable cost, 3) services are provided without being judgemental and 4) services are provided in separate rooms for adolescents to ensure privacy and confidentiality’. Each of these options represents an indicator of adolescent responsiveness. Every option chosen was given a score of 1 and a summary score was calculated to reflect understanding of adolescent responsiveness (range: 0–4).

### Data analysis

The data analysis was conducted using IBM SPSS version 23 (IBM Corp, Armonk, NY). Data was cleaned and all incomplete entries were removed before the analysis.We calculated descriptive statistics for HCWs responses regarding the availability of SRHS in their facilities, SRHS provided to adolescents and delivery of ASRHS, perceived adequacy of facilities in meeting the adolescents’ needs and understanding of adolescent responsive SRHS.

Subsequently, we ran univariable (not shown) and multivariable logistic regression analyses to assess associations between the demographic and professional characteristics of HCW and the provision and delivery of ASRHS. We also conducted univariable (not shown) and multivariable logistic regression analyses to assess associations between the perceived adequacy of the facilities of HCW in meeting the needs of adolescents and the demographic and professional characteristics of HCW as well as the specific ASRHS provided. We conducted univariable (not shown) and multivariable linear regression analyses to assess associations between the demographic and professional characteristics of HCW and their understanding of adolescent responsiveness. For all analyses of covariates, the level of significance was set at *p* ≤ 0.05.

## Results

### Descriptive findings

Participants were mostly women (66.3%), above the age of 40 years (53.1%) and married (89.5%); nearly all (94.9%) did not have a university degree (94.9%). Also, the majority were nurses or CHEW (79.5%). The others (20.5%) were environmental health officers, laboratory technicians or volunteers. Just over (53.6%) had 1–15 years working experience, nearly all (90%) had never had any training on adolescent SRH (for a full overview of assessed participant characteristics, see Table 1).

**Table 1:** Participants demographic and professional characteristics (n = 409)

	Number	Percentage
*Age group (years)*		
≤ 40	192	46.9
> 40	217	53.1
*Gender*		
Male	138	33.7
Female	271	66.3
*Marital status*		
Married	366	89.5
Not married	43	10.5
*Educational level*		
University degree	21	5.1
No university degree	388	94.9
*Specialty( Cadre) of health care worker*		
CHEW/nurse	325	79.5
EHO/ Lab tech /volunteer	84	20.5
*Years of work experience*		
1–15	219	53.5
≥ 16	190	46.5
*Ever received ASRH training*		
Yes	41	10.0
No	368	90.0

Note: CHEW: community health extension worker;

EHO = environmental health officers; Lab tech = laboratory technician

Most participants (89.2%) reported that some SRHS were available in their facilities. The most frequently mentioned SRHS available was antenatal care and delivery services (69.2%), followed by STI treatment (53.6%). Only 6.9% of the HCW indicated that STI testing was available in their facilities and treatment of STI was mostly based on symptoms and not on testing. Only 25.4% of HCW indicated they had contraceptives available in their health facilities, while merely 14.9% said post abortion care was available in their health facilities.

ASRHS most frequently provided was counselling, predominantly on abstinence (67%). Only 31.5% of the HCWs indicated that they provided counselling to adolescents on SRH issues other than abstinence, STI treatment was provided to adolescents by 53.6% and 15.2% provided post abortion care.

According to the HCW, 17.6% of the facilities did not provide any of the four SRHS for adolescents stipulated in the national policy (i.e., counselling, contraception, STI/HIV testing/treatment and post abortion care), 44% provided one service, 27.2% provided two services, 10% provided three services and only 1.2% provided all four services. Also, most participants (71.2%) said their facility did not provide SRHS without parental consent and 32.8% provided ASRHS in separate rooms. Their facilities were perceived as adequate in meeting the SRH needs of adolescents by 58.4% of the HCWs. Regarding understanding of adolescent responsive SRHS, 17.8% of participants did not mention any, 79.7% mentioned only one, 1.7% mentioned two, and 0.7% mentioned three. None correctly identified all four aspects of adolescent responsiveness.

### Covariate testing

Multivariable logistic regression analyses showed no significant association between the demographic and professional characteristics and SRHS provision, except for HCW gender, males were more likely to provide STI treatment (AOR = 1.538; CI = 1.01–2.33; p = 0.046) (for an overview, see Table 2).

**Table 2:** Multivariable logistic regression analyses of covariates of HCWs’ delivery of SHRS to adolescents (n = 409)

	Providing counselling			Providing contraceptives			Providing STI treatment			Providing post-abortion care			Providing ASRHS in separate room			Providing ASRHS without parental consent		
	AOR	95% CI	p-value	AOR	95% CI	p-value	AOR	95% CI	p-value	AOR	95% CI	p-value	AOR	95% CI	p-value	AOR	95% CI	p-value
*Age* group(years)																		
≤ 40	0.79	0.47–1.32	0.364	1.26	0.75–2.11	0.380	0.95	0.58–1.54	0.830	0.99	0.50–1.95	0.982	0.85	0.51–1.43	0.548	0.78	0.46–1.34	0.366
> 40	1			1			1			1			1		1	1		
*Sex*																		
Male	0.98	0.63–1.53	0.936	1.13	0.72–1.77	0.593	1.538	1.01–2.33	0.046*	0.84	0.46–1.52	0.564	0.99	0.64–1.55	0.99	0.91	0.57–1.45	0.694
Female	1			1			1			1			1		1	1		
*Marital status*																		
Married	0.85	0.42–1.74	0.657	0.84	0.41–1.75	0.644	0.82	0.42–1.60	0.551	0.68	0.29–1.57	0.371	0.72	0.36–1.43	0.342	0.80	0.39–1.66	0.557
Not married	1			1			1			1			1		1	1		
*Educational level*																		
Non degree	1.26	0.49–3.25	0.636	1.01	0.38–2.67	0.990	0.47	0.18–1.25	0.128	0.74	0.22–2.44	0.621	2.06	0.70–6.04	0.188	1.78	0.60–5.25	0.296
Degree	1			1			1			1			1		1	1		
*Specialty (Cadre) of health worker*																		
CHW/Nurses	0.65	0.37–1.13	0.127	1.38	0.82–2.33	0.229	1.48	0.89-2.45	0.128	0.88	0.45–1.73	0.709	0.72	0.43–1.22	0.227	0.72	0.42–1.23	0.227
Others*	1			1			1			1			1		1	1		
*Years of experience*																		
1–15	0.93	0.55–1.54	0.759	0.99	0.59–1.65	0.958	1.46	0.89–2.37	0.131	1.33	0.67–2.65	0.417	0.62	0.37–1.04	0.071	0.64	0.37–1.08	0.094
≥ 16	1			1			1			1			1		1	1		
*ASRH training*																		
Yes	0.93	0.46–1.86	0.832	0.83	0.41–1.69	0.612	0.79	0.40–1.56	0.501	1.71	0.75–3.92	0.205	1.40	0.69–2.82	0.35	1.49	0.73–3.06	0.273
No	1			1			1			1			1		1	1		

Note: CHW = community health worker; others = EHO and Lab tech

Furthermore, multivariable logistic regression analysis showed that HCW’s perception of their facilities being adequate in meeting the SRH needs of adolescents was significantly associated with the SRHS provided, notably the delivery of post abortion care (AOR = 3.612; CI = 1.886–6.917; *p* = 0.001) and providing SRHS to adolescents without parental consent (AOR = 3.067; CI = 0.125–0.854; *p* = 0.023) (for an overview, see Table 3).

**Table 3:** Multivariable logistic regression analyses of covariates of HCWs’ perceived adequacy of SRHS provided in meeting the SRH needs of adolescents (n = 409)

	AOR	95% C. I.	p-value
*Age group(years)*			
≤ 40	1.543	0.905–2.632	0.111
> 40	1		
*Gender*			
Male	1.154	0.729–1.828	0.540
Female	1		
*Marital status*			
Married	1.477	0.721–3.026	0.286
Not married	1		
*Educational status*			
Non-degree	2.050	0.755–5.562	0.159
Degree	1		
*Specialty (Cadre) of health provider*			
CHW/Nurse	0.993	0.574–1.719	0.980
Others	1		
*Years of working experience*			
1–15 yrs.	0.407	0.238–0.698	0.001*
≥ 16 yrs.	1		
*Ever trained on ASRH*			
Yes	0.929	0.442–1.953	0.846
No	1		
*Providing counselling*			
Yes	0.965	0.590–1.577	0.887
No	1		
*Providing contraceptives*			
Yes	0.929	0.571–1.511	0.767
No	1		
*Providing STI treatment*			
Yes	0.944	0.596–1.496	0.807
No	1		
*Providing post-abortion care*			
Yes	3.612	1.886–6.917	0.001*
No	1		
*Providing services* *In separate rooms*			
Yes	1.042	0.423–2.567	0.929
No	1		
*Providing SRH services without parental consent*			
Yes	3.067	0.125–0.854	0.023*
No	1		

Multivariable linear regression analysis showed that understanding of adolescent responsive SRH was better among HCW who were younger (β=-0.117), male (β = 0.074), unmarried (β=-0.008), nurses/CHEW (β = 0.032), held a university degree (β = 0.010), had more years of work experience (β = 0.068) and had received training on ASRH (β=-0.008). Understanding of adolescent responsiveness was also higher among HCWs who reported providing counselling (β = 0.067), contraceptives (β = 0.025) and not requiring parental consent (β = 0.275). In contrast, understanding of adolescent-responsiveness was lower amongst HCW who reported providing STI treatment (β=-0.033). (See Table 4)

**Table 4:** Multivariable linear regression analyses of covariates of HCWs understandings of adolescent responsive SRH

	B	SE B	β
*Age group(years)*	− 0.105	0.054	− 0.117
*Gender*	0.071	0.047	0.074
*Marital status*	− 0.011	0.075	− 0.008
*Educational status*	0.020	0.104	0.010
*Specialty (Cadre) of health provider*	0.035	0.057	0.032
*Years of working experience*	0.062	0.055	0.068
*Ever trained on ASRH*	− 0.014	0.076	− 0.009
*Providing counselling*	0.064	0.051	0.067
*Providing contraceptive*	0.024	0.051	0.025
*Providing STI treatment*	− 0.030	0.048	− 0.033
*Providing post abortion care*	0.083	0.066	0.066
*Providing ASRHS without parental consent*	0.274	0.098	0.275
*Providing ASRHS in separate rooms*	− 0.374	0.094	− 0.389
*R* ^*2*^		0.066	
*Adjusted R* ^*2*^		0.035	
* F for change in R* ^*2*^		2.141	

*Gender: 1-Male, 2-Female; Marital status:married-1, not married-2; Age group:≤40 − 1, > 40 − 2; Educational status:Non-degree-1, Degree-2, ever trained: 1-yes, 2- No; Type of counselling: 0- abstinence only, 1- contraceptives; providing contraceptive: 0-No, 1- Yes; provide STI treatment: 0-No, 1- Yes; provide post abortion care: 0-No, 1- Yes; providing ASRHS without parental consent: 0-No, 1-Yes; Providing ASRHS in separate rooms: 0-No, 1-Yes*

## Discussion

Nigeria has recently made efforts to ensure that adolescents have access to high quality and responsive SRHS, by developing a national policy and integrating SRHS into primary healthcare facilities. However, one frequently cited hindrance to utilization of SRHS by adolescents is the non-responsive attitude of HCWs [25–27]. We found that few of the HCWs in this study had a university degree, they were mostly nurses and CHEWs and in some cases – due to shortage of qualified personnel –laboratory technicians, environmental health officers, or volunteers with no health-related training. Also, most HCW had not received any prior training on ASRH, which is in line with findings from other African countries [32–34] and is likely to negatively affect the quality and outcome of ASRHS.

One fundamental requirement to providing adolescent responsive SRHS is the capacity of the providers. It is expected and stipulated in the Nigerian policy, that providers should be qualified and trained to provide evidence-based services, especially due to the sensitive nature of the matter for young people [30, 33, 35]. Although the basic qualifications expected at this level of healthcare in Nigeria are nursing and CHEW, having knowledgeable healthcare workers with additional training and competencies related to SRH is of added value [33, 35, 36]. Indeed, being competent in providing healthcare to adolescents is a vital skill acquired through a combination of pre-service education and in-service training for HCWs which should increase the likelihood of their ability to provide adolescent responsive SRHS [36, 37].

Most HCWs reported SRHS to be available in their facilities and the most frequently mentioned services were antenatal and delivery care, followed by STI treatment– which was generally based on symptoms with no testing. Nonetheless, these services did not cater for the range of adolescent SRH needs and were not adolescent focused [38, 39]. The SRHS mostly provided to adolescents was counselling on abstinence. This leaves other important SRH issues unaddressed, notably safe sex negotiation as well as the use of condoms and other contraceptives [38, 39]. We also found that not all the expected SRHS for adolescents were provided, despite the national policy stipulating that SRHS be made available and accessible to adolescents in an environment that is acceptable to them. Our results showed that, nearly half of the HCW provided only one of the four recommended ASRHS (counselling, contraceptives, STI/HIV testing and treatment, and post abortion care), while just over a quarter provided only two services and one in ten provided three services. Hardly any HCW reported providing all four services. Limited provision of SRHS for adolescents is a common finding reported in research, especially in African countries [40–42].

SRHS delivery is considered to be adolescent responsive when the recommended SRHS are provided without being judgmental and in an environment that respects the privacy and confidentiality of adolescents. We found that almost all HCWs who provided SRHS to adolescents, including those who provided STI treatment and post-abortion care, often did not ensure adolescents’ privacy by providing services in a separate room where they could not be seen and heard by other health service attendees. Also, HCW generally provided ASRHS only with parental consent. Privacy and confidentiality are key aspects of adolescent responsive SRHS delivery because adolescents do not appreciate discussing their sexual and reproductive health concerns in the presence of adults, including their parents [43, 44]. The World Health Organization (WHO) has concluded that SRHS are usually unacceptable to adolescents and not utilized if offered in such a way that confidentiality is not ensured [45–47].

We observed that the delivery of ASRHS was not significantly associated with any of the demographic and professional characteristics of HCWs, except for gender, with men being more likely to provide STI treatment. Having ever received training on ASRH was not significantly associated with providing any of the SRHS. However, this finding may be due to the small number of HCWs in our sample that had received such training. Therefore, these results should not be taken to suggest that training does not contribute to the ability of HCW to provide adolescent responsive SRHS, especially since other studies have highlighted that training resulted in more favourable attitudes towards providing ASRHS [36, 48, 49]. Rather, we take the low rate of HCWs who had received training in ASRHS delivery as a reminder of the importance that the delivery of such training is scaled-up.

In view of the limited provision of ASRHS reported by HCW and the non-adolescent responsiveness of ASRHS provision, it is noteworthy that some HCW felt their facilities adequately met the SRH needs of adolescents. Reflecting the national policy, HCWs who perceived adequacy in meeting the needs of adolescents were also more likely to offer recommended services, notably, post-abortion care and to provide SRHS without parental consent. It should be noted that provision of these ASRHS is contested, reflecting religious and personal beliefs that may affect ASRHS. Provision of SRHS is already generally rare, and HCW normally do not provide such services to adolescents, believing they are minors and must have the approval of their parents or guardian before seeking care [30, 31, 47]. Our findings especially raise concerns about the understanding of adolescent-responsiveness among HCW. We assessed four aspects that indicate understanding of adolescent responsive SRHS and found that only few HCW were able to mention even one of the aspects that constitute adolescent responsiveness and most HCWs did not demonstrate sufficient understanding of adolescent responsiveness. This is consistent with other studies that also reported poor knowledge of healthcare workers regarding appropriate ASRH and its importance for the delivery of quality services [50–53].

## Strength and limitations

To the best of our knowledge, this study it is the first to assess the extent of implementation of the Nigeria national policy on the responsive delivery of SRHS for adolescent and young people. Furthermore, the focus on HCWs perspectives of adolescent responsive SRHS delivery is innovative and provides novel insights into some of the possible reasons for the poor delivery of ASRH. The findings go beyond what is already known and offer guidance for policy and programmatic redirection. Additionally, we made use of a robust sampling frame and stepwise approach to recruit a sample of HCWs to optimally reflect the diversity of these professionals in Plateau State, Nigeria. We also acknowledge the limitations of our study; including the cross sectional design, which precludes any causal inferences. Furthermore, the study was conducted only in one state in Nigeria, limiting the generalizability of the findings to the entire country or other countries in the region. Also, data were collected through an interviewer-administered self-reported questionnaire, and responses may have been affected by recall bias as well as social desirability bias.

## Conclusion

Delivery of adolescent responsive SRHS in Plateau State, Nigeria is not as set out in the national policy. This study shows that most HCW at PHC do not deliver the full range of ASRHS and do not deliver ASRHS in an adolescent responsive manner. Encouragingly, the perception of HCW regarding their services being adolescent responsive was higher when they actually delivered relevant ASRHS. In general, the HCWs in this study, however, had poor understanding of adolescent responsive SRHS. To ensure delivery of adolescent responsive SRHS, we recommend scaling up appropriate training for HCW in Plateau State to improve their knowledge and skills in providing quality SRHS to adolescents who need it. However, although knowledge and training are essential, our results also indicate that this is likely not sufficient to provide responsive ASRHS in the absence of structural facilitating factors, such as private consulting rooms, which help ensure that adolescents experience a sense of safety and privacy to freely discuss SRH issues.

## Electronic supplementary material

Below is the link to the electronic supplementary material.

Supplementary Material 1

## Data Availability

All data generated or analysed during this study are included in this published article.
