# Supplementary material for: Healthcare workers’ delivery of adolescent responsive sexual and reproductive healthcare services: an assessment in Plateau state, Nigeria
Source: BMC Womens Health. 2023 Mar 25;23:132. doi: 10.1186/s12905-023-02288-1 (PMC10040103; doi:10.1186/s12905-023-02288-1)
Supplement: Supplementary file 1 — Supplementary Material 1 [file 12905_2023_2288_MOESM1_ESM.docx]

QUESTIONNAIRE FOR HEALTH CARE PROVIDERS

BIODATA

Name of facility…………………………………………………………

LGA----------------------------------------------------------------------

Cadre of health provider-----------------------------------------------

Educational status------------------------------------------------------

Years of experience----------------------------------------------------

Duration of working in present facility-------------------------------

Age in years-----------------------------------------------------------

Sex---------------------------------------------------------------------

Marital status----------------------------------------------------------

AVAILABILITY OF SRH SERVICES

| Does this facility have SRH services for adolescents | Yes | No |  |  |  |
| --- | --- | --- | --- | --- | --- |
| Which of these SRH services are available | ANC/delivery | Contraceptive | STI/HIV   1. Testing and treatment 2. Treatment only | Post abortion care | Others specify |
| Are these services specific for adolescents | Yes | No | Other ( specify) |  |  |
| Do existing services in your facility meet the needs of adolescents? | Yes | No |  |  |  |

DELIVERY OF SRH SERVICES

| **Questions** |  |  |  | **others**  **specify** |
| --- | --- | --- | --- | --- |
| What counseling do you give an adolescent on prevention of pregnancy? | Abstenance  alone | Use contraceptives | Use condom | Others specify |
|  |  |  |  |  |
| What counseling do you give an adolescent on prevention of STI/HIV? |  |  |  |  |
| What counseling do you give an adolescent on GBV? | Avoid unsafe places | Respect for males and females | Report cases of abuse | Others specify |
|  |  |  |  |  |
| What sexual and reproductive health services would you offer adolescents who does not want to get pregnant? | Abstenance  alone | contraceptives | condoms | Others specify |
| What sexual and reproductive health services would you offer adolescents who does not want to be infected with STI? |  |  |  |  |
|  |  |  |  |  |
| Are there ever any exceptions to the services for adolescents? If so, explain | Yes | No | Others  specify |  |
|  |  |  |  |  |

|  | Yes | No |  | Others specify |
| --- | --- | --- | --- | --- |
| Are contraceptive offered? |  |  |  |  |
| Are condoms provided to both males & females? |  |  |  |  |
| Is pregnancy testing offered to adolescents? |  |  |  |  |
| Is STI testing available for adolescents? |  |  |  |  |
| Is STI treatment available for adolescents? |  |  |  |  |
| Is post abortion care available for adolescents? |  |  |  |  |
| Is Antenatal care, delivery and postnatal care available for adolescents? |  |  |  |  |
| Would you offer contraceptives to adolescents? |  |  |  |  |
| What will be your reason for not offering contraceptive services to adolescents? |  | | |  |

|  | Yes | No | Others specify |  |
| --- | --- | --- | --- | --- |
| Would you provide privacy and respect to any adolescent in attending to them |  |  |  |  |
| Would you require parental consent before attending to adolescents? |  |  |  |  |
| Would you involve adolescents in any of the services delivered? |  |  |  |  |
| If you haven’t involved youth in decision-making, what are some reasons why you haven’t? |  | | |  |

TRAINING / KNOWLEDGE

| Have you ever been trained on ASRH? | **Yes** | **No** | **Others**  **Specify** |  |
| --- | --- | --- | --- | --- |
| If yes by whom? |  |  |  |  |
| What makes health services adolescent friendly? | When SRH services are available | When services are available and accomodating to adolescents | When environment is friendly | Others specify |
| When is delivery of SRHS adolescent responsive? | When all SRHS are made available and accessible to adolescents | when the services are provided free or at affordable cost, | When services are provided without being judgemental, and | When services are provided in separate rooms for adolescents to ensure privacy and confidentiality’ |

THANK YOU
